# Comparative performance of traps in catching tsetse flies (Diptera: Glossinidae) in Tanzania

**DOI:** 10.4102/ojvr.v83i1.1057

**Published:** 2016-06-23

**Authors:** Imna I. Malele, Johnson O. Ouma, Hamisi S. Nyingilili, Winston A. Kitwika, Deusdedit J. Malulu, Henry B. Magwisha, Eliningaya J. Kweka

**Affiliations:** 1 Vector & Vector Borne Disease Institute, Tanga, Tanzania; 2 Biotechnology Research Institute, Kenya Agricultural and Livestock Research Organization, Muguga, Kenya; 3 Africa Technical Research Centre, Vector Health International, Arusha, Tanzania; 4 Vector & Vector-Borne Diseases Centre, Kigoma, Tanzania; 5 Tanzania Veterinary Laboratory Agency, Dar Es Salaam, Tanzania; 6 Division of Livestock and Human Diseases Vector Control, Tropical Pesticides Research Institute, Arusha, Tanzania; 7 Department of Medical Parasitology and Entomology, Catholic University of Health and Allied Sciences, Mwanza, Tanzania

## Abstract

This study was conducted to determine the efficiency of different tsetse traps in 28 sites across Tanzania. The traps used were biconical, H, NGU, NZI, pyramidal, S3, mobile, and sticky panels. Stationary traps were deployed at a distance of 200 m apart and examined 72 h after deployment. The results showed that 117 (52.2%) out of the 224 traps deployed captured at least one *Glossina* species. A total of five *Glossina* species were captured, namely *Glossina brevipalpis, Glossina pallidipes, Glossina swynnertoni, Glossina morsitans*, and *Glossina fuscipes martinii*. Biconical traps caught tsetse flies in 27 sites, pyramidal in 26, sticky panel in 20, mobile in 19, S3 in 15, NGU in 7, H in 2 and NZI in 1. A total of 21 107 tsetse flies were trapped, with the most abundant species being *G. swynnertoni* (55.9%), followed by *G. pallidipes* (31.1%), *G. fuscipes martinii* (6.9%) and *G. morsitans* (6.0%). The least caught was *G. brevipalpis* (0.2%). The highest number of flies were caught by NGU traps (32.5%), followed by sticky panel (16%), mobile (15.4%), pyramidal (13.0%), biconical (11.3%) and S3 (10.2%). NZI traps managed to catch 0.9% of the total flies and H traps 0.7%. From this study, it can be concluded that the most efficient trap was NGU, followed by sticky panel and mobile, in that order. Therefore, for tsetse fly control programmes, NGU traps could be the better choice. Conversely, of the stationary traps, pyramidal and biconical traps captured tsetse flies in the majority of sites, covering all three ecosystems better than any other traps; therefore, they would be suitable for scouting for tsetse infestation in any given area, thus sparing the costs of making traps for each specific *Glossina* species.

## Introduction

There are 33 species and subspecies of tsetse flies (Diptera: Glossinidae) in Africa, infesting 36 countries south of the Sahara (Gooding & Krafsur 2005). Tsetse flies transmit trypanosomes that cause African trypanosomiasis (AT) in humans and livestock. The disease has negative effects on the hosts as it causes low productivity, leading to mortality when untreated. In livestock, some of the direct losses caused by the disease include abortion, reduced milk yield and poor calf crop. Given its role in transmitting AT in livestock and humans, the tsetse fly deserves special attention, particularly with regard to its response to different sampling methods in a variety of ecological settings. Studies on tsetse ecology and control have mainly relied on the availability of an efficient sampling method, a number of which have been described. The main sampling methods include the use of fly nets and fly rounds (Pollock 1982), sticky materials (Vreysen, Khamis & Van der Vloedt 1996), and fabric traps (Challier & Laveissiere 1973; Dransfield & Brightwell 1997; Ndegwa & Mihok 1999; Vale 1982; Vale, Flint & Hall 1986). Most of the traps used in the foregoing studies were developed in west and southern Africa for riverine and savannah tsetse species, respectively. Interestingly, the sampling studies carried out thus far have revealed variable efficiency of the traps in capturing tsetse flies. In view of this, the efficiency of traps to capture certain tsetse species has been enhanced through modification of various designs of traps for use against particular target species in relation to the environment (Ndegwa & Mihok 1999; Ndegwa, Mihok & Oyieke 2001).

Traps basically function through visual stimuli. In the field, however, the visual stimuli can be greatly obstructed by vegetation, particularly for forest species. In such cases, attraction of flies to traps is enhanced through the use of odour attractants. Three groups of natural odours have been determined so far from the host animals: those found in urine (e.g. phenols), breath (e.g. acetone) and skin secretions (e.g. octenols (Vale 1993). The response of tsetse flies to particular odours varies amongst species (Green 1986; Kuzoe & Schofield 2004).

Principally, tsetse traps are made up of blue and black textile materials and white netting. The blue colour has been found to be a visual stimulus or attractant to flies. Field observations on tsetse trapping show that all tsetse traps attract tsetse flies to land on them. However, the difference in their relative trapping efficiency is based on their designs, especially the entrance for flies into the retaining cage. This has been the basic reason for modifying conventional traps and designing new ones to suit the target species in different types of habitats. Considerable advances have been made in the development of efficient traps for tsetse flies, as trapping is increasingly being used for population suppression and control of tsetse flies. Currently, the common traps used for sampling and monitoring economically important tsetse species include the biconical trap (Challier *et al.* 1977), developed for sampling *Glossina morsitans*; F3 and Epsilon (Flint 1985; Green & Flint 1986) for *Glossina pallidipes*; NGU (Dransfield & Brightwell 1997) for *G. pallidipes*; NZI (Mihok 2002) for tsetse flies, horse flies, deer flies and stable flies; S3 (Ndegwa & Mihok 1999) for *Glossina swynnertoni* and the pyramidal (Goutex & Lancien 1986) for *Glossina tachinoides*. However, it has been found that the difference in trap efficiency is related to the behavioural differences between the species and varies between different populations of the same species. In some cases, minor modifications are required to improve the efficiency of a trap. For example, the S3 trap underwent a series of modifications before it was perfected (Ndegwa & Mihok 1999). Knowledge of the response of particular tsetse species to specific traps with or without odours is important for enhancing the efficiency of tsetse fly suppression operations and the formation of barrier systems used in tsetse control or eradication campaigns.

In Tanzania, despite the vast area that is infested by tsetse flies, tsetse trapping has mainly depended on traps developed outside the country, targeting different vegetation types and tsetse species, except for the mobile, scoop (Kuzoe & Schofield 2004) and sticky panel (Vreysen *et al.* 1996). The dominant tsetse species are the savannah tsetse species, which include *G. pallidipes, G. swynnertoni* and *G. morsitans morsitans.* Other species that are not widely distributed include *Glossina austeni, Glossina brevipalpis, Glossina longipennis, Glossina fuscipes martinii* and *G. fuscipes fuscipes*. Unpublished results (VVBD – Tanga) show that the response of tsetse flies to traps in Tanzania varies from one area to another even within the same species. It is likely that such variation could affect the results obtained from tsetse surveys, particularly data on fly density and distribution. This study was aimed at investigating the efficiency of different traps for different tsetse species so that, if need be, a single trap could be used for sampling different tsetse species if it proved to be efficient against several tsetse species. For example to trap *G. swynnertoni*, several traps have been used, including pyramidal traps (Malele *et al.* 2007; Mramba *et al.* 2013), Epsilon traps (Auty *et al.* 2012), rectangular cloth targets and small leg panels (Mramba *et al.* 2013). However, the comparative performance of several traps against the species has not been documented. Variations in response in relation to traps and fly species have a negative impact, especially when planning for tsetse and trypanosomiasis control. Furthermore, such variations could lead to underestimation of the production losses and public health harm caused by tsetse flies and tsetse-borne diseases. On the other hand, if one trap is found to be efficient against several tsetse species, then the cost of making several traps to suit several species present in an area could be avoided. Apart from sampling, efficient tsetse sampling traps have been used elsewhere as cheap control devices against the vector (Madubunyi 1988).

We report the results of studies carried out to determine the relative efficiency of different tsetse traps in trapping various species of tsetse flies in different ecological settings in the Serengeti ecosystem (Mara region), the western ecosystem (Kigoma and Tabora regions) and the southern ecosystem (Selous Game Reserve, which covers Lindi and the south-eastern part of the Morogoro region), and we recommend the most efficient trap for each species according to the ecological zonation in the three ecosystems in Tanzania. The data presented were collected from 2008 to 2012.

## Materials and methods

### Study areas

#### Serengeti ecosystem

The Serengeti ecosystem is situated in the Mara region, northern Tanzania, and consists of Serengeti National Park (SENAPA), Ngorongoro Conservation Area and Maswa Game Reserve. The site comprises a savannah habitat with a wide range of wild game, including wildebeests, elephants, antelopes, lions, wild pigs, buffaloes and giraffes, most of which serve as a source of blood meals for tsetse. Tsetse fly species found in the Serengeti include *G. morsitans, G. pallidipes, G. brevipalpis* and *G. swynnertoni*, with the predominant species being *G. swynnertoni.*

#### Western ecosystem

**Uvinza (Kigoma):** The Uvinza site is found in western Tanzania and borders the Moyowosi Game Reserve to the west and Mpanda/Uvinza Game Reserve and Ilunde and Chakulu Forest Reserves to the east. The habitat is mainly miombo woodland. The wild animals found at Uvinza include buffaloes, antelopes and wild pigs migrating from the neighbouring game reserves. However, part of the area is used for cattle ranching. The common tsetse flies in the Uvinza area are *G. morsitans, G. pallidipes, G. f. martinii* and *G. brevipalpis*.

**Ugalla (Urambo):** The Ugalla Game Reserve is the key component of the study in the Ugalla area. The climate is defined by a distinct wet season from December to June and a dry season from July to November. The vegetation consists of dry Zambezian miombo woodland; wooded grassland with reduced tree cover is the most widespread vegetation type in the area. Wild animals are common in the ecosystem. The herbaceous layer is dominated by *Hyperrhenia* species, with a shrub layer of saplings of the canopy trees. The livelihoods of the local people around Ugalla Game Reserve consist of a mixture of activities such as livestock keeping, agriculture, fishing, hunting, beekeeping and the harvesting of forest products (Lutabingwa 2006). Tsetse sampling was conducted at Kangeme, Lumbe, Ukumbi-Siganga and Usinga. Common tsetse species in the area are *G. morsitans* and *G. pallidipes.*

#### Southern ecosystem

**Selous Game Reserve:** The park varies from rolling grassy woodlands and plains to rocky outcrops cut by the Rufiji River – the lifeblood of the park, with tributaries that form a network of lakes, lagoons and channels. This is one of the areas in Tanzania with a high density of wild animals that include, naming but a few, antelopes, crocodiles, hippos, and black-and-white colobus monkeys in the riverine forests. During the dry season from June to October, animals tend to concentrate along the river linked to the Rufiji in Lake Tagalala, where waterbuck, reedbuck and bushbuck are abundant. In the dry season, there is a notable migration of elephants between the Selous Game Reserve and Mozambique’s Niassa Game Reserve. Tsetse sampling was conducted along game-viewing and camping sites owned by Mivumoni River Lodge and Selous Luxury Camp of Serena Hotels. Common tsetse species include *G. morsitans* and *G. pallidipes* (Malele *et al.* 2013).

### Traps

Eight traps, namely the biconical, pyramidal, NGU, mobile, sticky panel, S3, NZI and H, were compared for relative efficiency in trapping different tsetse fly species in a total of 28 sites in the three ecosystems (Serengeti, western and southern Tanzania). Deployment of the traps in the field was as described by Vale (1982) and FAO (1992). The traps were deployed at an interval of about 200 m apart in a Latin square study design (days × treatments × sites) and remained at one site for 72 h before being transferred to a new site. Tsetse flies caught in each trap at each site were identified to sex and species levels, pooled and recorded.

Tsetse catches by the sticky panel and mobile traps were included in the analysis just for comparison purposes because any fly that lands on sticky panels is retained and those trapped by a mobile trap are scooped (sucked into a retaining cage), whereas tsetse flies may visit and leave other stationary traps without entering the retention cage.

### Data analysis

Data on the tsetse catches from the 28 sites of the three ecosystems were collected and recorded on sheets, entered in Microsoft Excel and then transferred to Epi Info (2014) analytical software for analysis. One-way analysis of variance was used to analyse the efficiency of each trap for the five species trapped. Tsetse fly counts were used as independent variables and trap type, species and ecosystem as grouping variables. The overall comparison of the traps’ sampling efficiency regardless of the tsetse fly species was done using generalised linear model univariate analysis. Traps and sexes were considered for a full factorial model for main-effect analysis. Separation of means was done at the 95% confidence interval and a significance level of 5% in all the statistical tests.

## Results

### Overall counts of *Glossina* species from various areas

Overall, a total of 21 107 tsetse flies were caught from the 28 sites (Table 1). Six sites with the highest counts contributed 12 358 tsetse flies (58.5%); these were Death Valley, 5928; Uvinza, 1661; Hippo Area, 1298; Hembe, 1289; Retima Pool, 1101 and Mareo, 1081. The remaining 8749 flies were caught at the remaining 22 sites.

**TABLE 1:** Descriptive statistics for sites.

Ecosystem	Site	Number of traps	Total catches	Mean	s.d.	Total flies per ecosystem
Serengeti	Banagi	8	455	56.88	34.56	16 312
	Bilila	8	53	6.63	0.97	
	Death Valley	8	5928	741.00	518.31	
	Hembe	8	1289	161.13	108.28	
	Hippo Area	8	1298	162.25	109.07	
	Ikoma Gate	8	196	24.50	11.67	
	Kilima Fedha	8	452	56.50	34.29	
	Kiongore	8	41	5.13	2.03	
	Kubukubu	8	374	46.75	27.40	
	Makao	8	553	69.13	43.22	
	Makoma Hill	8	1	0.13	5.57	
	Mareo	8	1081	135.13	89.89	
	Mbala Gate	8	319	39.88	22.54	
	Mbuzi Mawe	8	346	43.25	24.93	
	Okoma Gate Ws	8	460	57.50	35.00	
	Retima Pool	8	1101	137.63	91.66	
	Romoti R	8	320	40.00	22.63	
	Serena Lodge	8	589	73.63	46.40	
	Seronera	8	279	34.88	19.00	
	Sopa Lodge	8	579	72.38	45.52	
	Tunner Spring	8	598	74.75	47.20	
Western	Ugala	8	858	107.25	70.18	4390
	Urambo	8	672	84.00	53.74	
	Usinga	8	125	15.63	5.39	
	Gombe	8	840	105.00	68.59	
	Kagerankanda	8	234	29.25	15.03	
	Uvinza Malahi	8	1661	207.63	141.16	
Southern	Selous	8	405	50.63	30.14	405

The trapping performance of stationary traps for different *Glossina* species demonstrated that although the NGU traps caught tsetse in only 7 sites compared with the biconical and pyramidal traps, which caught flies in over 25 sites, NGU trapped more tsetse flies than any other traps used in the study. By ranking the means, NGU traps were found to be significantly more efficient (*p* < 0.05) than biconical and pyramidal traps (Table 2).

**TABLE 2:** Overall counts of positive traps for *Glossina* species.

Species	Biconical	H	Mobile	NZI	NGU	Pyramidal	S3	Sticky panel	Total	% Total
*Glossina brevipalpis*	4	0	30	0	0	2	0	0	36	0.2
*Glossina morsitans*	106	125	165	0	727	127	0	15	1265	6.0
*Glossina pallidipes*	503	30	186	0	4011	344	834	657	6565	31.1
*Glossina swynnertoni*	1705	0	2859	180	1623	2227	1326	1872	11 792	55.9
*Glossina fuscipes martinii*	73	0	0	0	494	52	0	830	1449	6.9

**Total**	**2391**	**155**	**3240**	**180**	**6855**	**2752**	**2160**	**3374**	**-**	**-**
**Means**	**478.2**	**31.0**	**648**	**36**	**1371.0**	**550.4**	**432.0**	**674.8**	**-**	**-**
**% Total catch per trap**	**11.3**	**0.73**	**15.4**	**0.85**	**32.5**	**13.0**	**10.2**	**16.0**	**-**	**-**

Five *Glossina* species were caught at the following proportional percentages in decreasing order: *G. swynnertoni* (55.9%), *G. pallidipes* (31.1%), *G. f. martini* (6.9%), *G. morsitans* (6.0%) and *G. brevipalpis* (0.2%). The average species count of *G. swynnertoni* was significantly different (*p* = 0.05) from that of all other species; *G. pallidipes* was significantly different from *G. morsitans, G. f. martinii* and *G. brevipalpis*, but the last three species were not significantly different from one another.

The performance of traps for each ecosystem is shown in Table 3. Nearly all the traps were able to catch flies in the Serengeti ecosystem, except for the H trap. Table 4 shows the occurrence of different tsetse species per ecosystem.

**TABLE 3:** Comparison of means of trap performance per ecosystem.

Traps	Ecosystems		

	Serengeti	Southern	Western
Biconical	42.7	3.5	49.0
H	0.0	0.0	12.2
Mobile	67.2	175.5	75.5
NGU	115.9	0.0	176.9
NZI	4.4	0.0	0.0
Pyramidal	57.5	8.0	15.1
S3	53.6	0.0	0.0
Sticky panel	44.6	7.5	63.5

**TABLE 4:** Mean rank of tsetse species per ecosystem.

Species	Ecosystems		

	Serengeti	Southern	Western
*Glossina pallidipes*	26.4	23.5	46.0
*Glossina morsitans morsitans*	0.0	25.1	39.8
*Glossina swynnertoni*	70.6	0.0	0.0
*Glossina brevipalpis*	36.0	0.0	0.0
*Glossina fuscipes martinii*	0.0	0.0	208.3

Out of the 224 traps deployed, only 117 (52.2%) captured flies. Table 5 shows that 73 (62.4%) out of these 117 traps caught a single *Glossina* species, whereas 42 (35.9%) traps caught two species each, and only 2 (1.7%) traps had three species each. The following traps demonstrated consistent efficiency, in decreasing order, for trapping *G. swynnertoni*: mobile, sticky panel, pyramidal, and biconical. Biconical traps caught tsetse flies at 27 sites, pyramidal at 26, sticky panel at 20, mobile at 19, S3 at 15, NGU at 7, H at 2 and NZI at 1.

**TABLE 5:** Single, double or triple species trapped by different traps.

Species	Biconical	H	Mobile	NGU	NZI	Pyramidal	S3	Sticky panel	Total	Occurrence of species	% Occurrence of species
*Glossina fuscipes martini*	1	0	0	1	0	1	0	1	4	73	62.4
*Glossina morsitans*	1	0	0	0	0	0	0	1	2		
*Glossina pallidipes*	1	1	0	2	0	3	0	2	9		
*Glossina swynnertoni*	10	0	17	0	1	11	6	13	58		
*Glossina pallipes*+ *Glossina fuscipes martinii*	1	0	0	1	0	1	0	1	4	42	35.9
*Glossina pallidipes*+ *Glossina morsitans*	3	1	1	1	0	2	0	0	8		
*Glossina pallidipes*+ *Glossina swynnertoni*	9	0	0	2	0	7	9	2	29		
*Glossina swynnertoni*+ *Glossina brevipalpis*	0	0	1	0	0	0	0	0	1		
*Glossina pallidipes*+ *Glossina swynnertoni*+ *Glossina brevipalpis*	1	0	0	0	0	1	0	0	2	2	1.7

**Total**	**27**	**2**	**19**	**7**	**1**	**26**	**15**	**20**	**117**	**-**	**-**

*Glossina swynnertoni* was captured by 90 traps, *G. pallidipes* by 52, *G. morsitans* by 10 and *G. f. martinii* by 8; the least trapped tsetse fly was *G. brevipalpis*, caught by 3 traps. *Glossina pallidipes* and *G. swynnertoni* were sympatric species recorded in 29 (24.8%) occurrences (Table 5).

Out of the 21 107 tsetse flies trapped, 1449 were trapped at Uvinza and Kagerankanda in Kigoma. *G. f. martinii* was only found in the western part of the country, along the lake shores of Lake Tanganyika and along the rivers draining into Lake Tanganyika. Most of the *G. f. martinii* flies were not sorted into their respective sexes. Of the sorted flies (19 658), females were significantly more numerous than males (one male to two females) (*p* < 0.05). Also, more females were trapped by sticky panels than by mobile traps, and more males were trapped by mobile traps than by sticky panels. In total, however, the mobile traps caught more flies than the sticky panels (Table 6).

**TABLE 6:** Mean tsetse sexes per mobile trap versus sticky panel.

Sex	Mobile catches	Sticky panel catches	*p*
Males	36.283	14.426	0.0500
Females	25.038	33.981	0.0000

**Total**	**61.321**	**47.148**	**0.2207**

## Discussion

Five species of *Glossina* were recorded in this study. Proportionally, the species ranged in decreasing order from *G. swynnertoni* (55.9%) to *G. pallidipes* (31.1%), *G. f. martinii* (6.9%), *G. morsitans* (6.0%) and *G. brevipalpis* (0.2%). *G. swynnertoni* was the dominant species in the Serengeti ecosystem, whereas *G. morsitans* was the dominant species in the western ecosystem. Species dominance in specific regions is likely due to the ready and continuous availability of preferred hosts in those regions. Confirmation of preferred hosts is only achievable through blood meal analysis, which was outside the scope of this study. Combining an analysis of tsetse-trapping efficiency with an analysis of the origin of the blood meals for some of the most commonly used tsetse traps would add value and is a subject for further investigation.

On trap performance, the NGU traps caught tsetse flies at only 7 sites compared with the biconical and pyramidal traps, which caught flies in over 25 sites; however, NGU trapped more tsetse flies than the rest of the traps used in the study. Although this demonstrates the superior efficiency of the NGU trap, it is unclear whether the failure to catch flies at most of the sites was a result of inter- and intra-species differences in behaviour and response to NGU.

All the traps used in this study, with the exception of H and NZI, which trapped very few flies, could be used for sampling tsetse flies in the Serengeti ecosystem, although their efficiency varies. In the western zone, all traps except NZI and S3 could be used for sampling or trapping flies. In the southern zone, the mobile, biconical, pyramidal and sticky panel traps could be used for sampling or trapping purposes. Again, *G. swynnertoni* could be trapped by all the traps used in the study except the H trap.

The performances of the sticky panel and mobile traps showed no significant difference in the proportion of flies caught. The catches were 16% by sticky panel and 15.4% by mobile trap. It was noted, however, that the mobile trap can be used to catch *G. swynnertoni, G. brevipalpis* and *G. morsitans*, whereas the sticky panel is more suitable for catching *G. swynnertoni.* The suitability of the sticky panel for catching *G. swynnertoni* is consistent with the observations of Mramba *et al.* (2013), although they used a different type of sticky panel, that is, all blue-legged panels. On the other hand, NGU performed better in catching *G. pallidipes, G. morsitans, G. swynnertoni* and *G. f. martinii*. In this study, NGU performed best for *G. pallidipes*, and this concurs with earlier findings for this trap, which was developed for savannah species (Dransfield *et al*. 1986).

The pyramidal trap has been the trap of choice in many studies in Tanzania because of its simplicity in deployment; however, its trapping ability was not as superior as NGU, although it still was able to trap about 12.4% of the total flies in this study, second to NGU (amongst stationary traps) and slightly better than the biconical trap, which is usually used as a sampling device (Takken 1984) (Table 6). The suitability of biconical as a sampling trap (unbiased towards any one species) was demonstrated by its being one of the traps that trapped nearly all tsetse species in the study sites (Table 5).

It has been documented that the H trap is good for trapping *G. brevipalpis* (Kappmeier 2000), which is typically a forest species. However, in this study the performance of the trap against *G. brevipaplis* was not good; perhaps vegetation cover could have influenced the results in the present study. Trapping was carried out mostly in savannah wooded areas and not in forested areas, where the species is mostly found.

**FIGURE 1:**
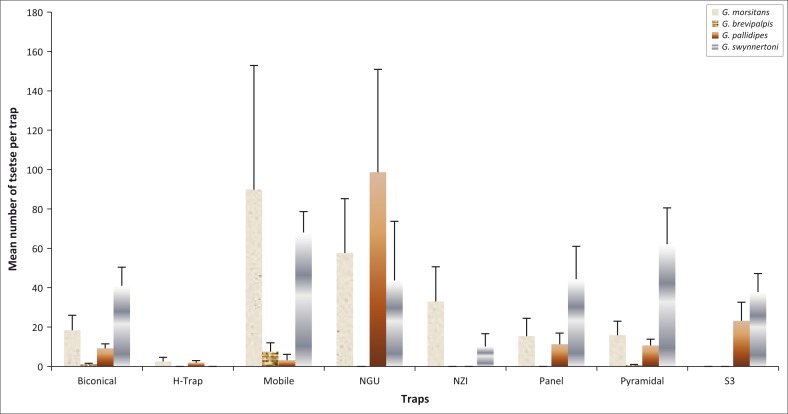
Trapping performance of different traps for different species of tsetse flies.

**FIGURE 2:**
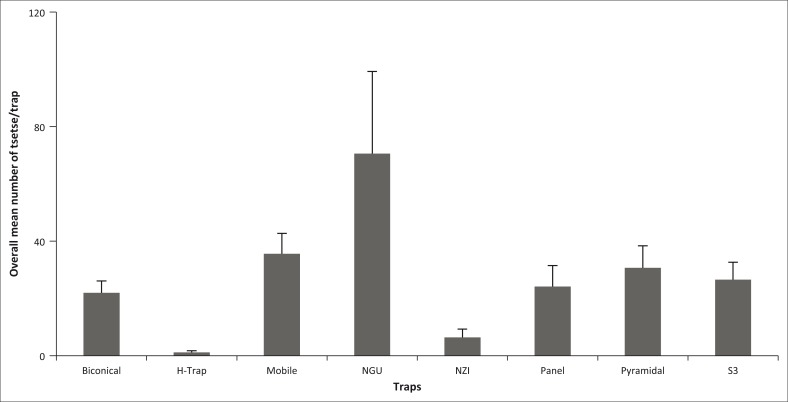
Overall tsetse fly species mean number per trap.

For *G. m. morsitans*, the mobile trap performed better, followed by NGU (Figure 1). When evaluating the overall performance of traps regardless of species, and by analysing the data considering the density of flies sampled regardless of species, significant statistical difference was found among the traps, with NGU having the highest sampling efficiency (Figure 2). This affirms the superior performance of NGU, as already discussed earlier. Only NGU trapped more flies as a stationary trap than the mobile trap, which sucks in any fly in the vicinity.

From the foregoing observations, it can be concluded that the different tsetse traps being used in Tanzania have varying trapping efficiencies, which in some cases seems to be dependent on the tsetse fly species being sampled and the ecological setting. However, pyramidal and biconical traps can be used for sampling or trapping tsetse flies, hence saving the cost of having different traps developed for each specific *Glossina* species.
